# The characteristics of patients who discontinue their dying process – an observational study at a single university hospital centre

**DOI:** 10.1186/s12904-015-0070-7

**Published:** 2015-12-07

**Authors:** Christian Schulz, Daniel Schlieper, Christiane Altreuther, Manuela Schallenburger, Katharina Fetz, Andrea Schmitz

**Affiliations:** Interdisciplinary Centre for Palliative Medicine, Medical Faculty, Heinrich Heine University Düsseldorf, Moorenstraße 5, 40225 Düsseldorf, Germany; Department of Health, Witten/Herdecke University, Alfred-Herrhausen-Straße 50, 58448 Witten, Germany; Department of Anesthesiology, Medical Faculty, Heinrich Heine University Düsseldorf, Moorenstraße 5, 40225, Düsseldorf, Germany

**Keywords:** Palliative care, Terminal care, Critical pathways, Quality of health care, Hospices, Diagnosis of dying, End-of-life care plan

## Abstract

**Background:**

End-of-life integrated care plans are used as structuring tools for the care of the dying. A widely adopted example is the Liverpool Care Pathway for the Dying Patient (LCP). Recently, several concerns were raised about LCP care, such as a worry that diagnosis of dying might be leading to a self-fulfilling trajectory, including hastening of death. However, data on rates of discontinuation of LCP care are lacking. In an observational study, we therefore investigated the incidence, features and trajectory of patients who were discontinued from the LCP. We hypothesised that (1) it is common to discontinue patients from the LCP, (2) quality of life does not decrease for discontinued LCP patients, and (3) discontinued patients live longer than patients who remain within LCP care.

**Methods:**

All adult patients who were diagnosed as dying in a German university hospital specialized palliative care unit were included in 2013 and 2014. Actuarial estimation of survival prognostication tools and a number of quality of life indicators were used for data collection. Survival time was analysed using Kaplan-Meier estimates. Group differences in quality of life were tested using multivariate analysis of variance.

**Results:**

159 patients were included in a digital version of the LCP. 15 patients (9.4 %) were discontinued later. Quality of life did not decrease for discontinued patients during LCP care (*p* = 0.16). LCP discontinued patients lived significantly longer than the remaining LCP subgroup (difference of means 296 hours, 95 % confidence interval 105.5 to 451.5 hours; difference of survival function estimates *p* < 0.0001).

**Conclusions:**

When patients are diagnosed as dying, death is not the inevitable outcome of an end-of-life integrated care plan such as the LCP. Instead, it is common to discontinue the LCP care. Regular careful interprofessional assessments are important for identifying those patients who need to be discontinued from their end-of-life care plan. In this study, we found no evidence for harm by the LCP. We conclude that a correctly applied integrated care plan can be useful to provide good and safe care for the dying.

**Trial registration:**

Internal Clinical Trial Register of the Medical Faculty, Heinrich Heine University Düsseldorf, No. 2015053680 (22 May 2015).

## Background

For dying patients, receiving the best possible quality of care is considered a basic human right [1]. An end-of-life care plan, such as the Liverpool Care Pathway for the Dying Patient (LCP) [2], is a complex intervention to structure the care of the dying in the last hours or days of life [3]. The original aim of the LCP was to transfer care practice from a hospice setting to other care settings [2]. The LCP was primarily meant to measure outcomes and to facilitate audit rather than to influence outcomes [2, 4, 5]. However, implementing interventions that assess the quality of life in palliative care does result in improved outcomes [6], and the use of an integrated care pathway promotes good practice. Therefore, it is conceivable that the LCP improves both the efficiency and quality of care and ensures good communication within the team and with patients and their relatives [7].

A recent Cochrane systematic review [8] found little evidence that an integrated care pathway during the dying phase improves outcomes: Only one cluster randomized trial showed that respect, dignity and kindness as well as control of dyspnoea were significantly improved by the LCP, while the overall quality of care was not significantly different [9]. However, this study was underpowered and may have missed outcome differences [9]. In non-controlled before and after studies [10], the use of LCP was shown to improve coordination of care [11], communication within the interdisciplinary team [12], symptom control [13], documentation [12, 13], use of appropriate medication [12, 14], bereavement levels of relatives [15], communication with patients or families [12, 16] and family support [11]. Focus group meetings of nurses and physicians indicate that the use of LCP strengthens the interprofessional teamwork [17, 18].

The LCP is used internationally in at least 22 countries [19]. In England, the LCP was recognized as a model of best practice in 2001 and was widely implemented in the following years [20, 21]. However, according to a commissioned report by Neuberger et al., the LCP was not applied properly in a number of places [22], and the LCP was phased out in the United Kingdom in 2014 [23, 24]. Notably, even the authors of this report, along with other commentators, stressed the point that if correctly implemented the LCP use resulted in patients dying a “peaceful and dignified death” [22, 25–31].

**Fig. 1:**
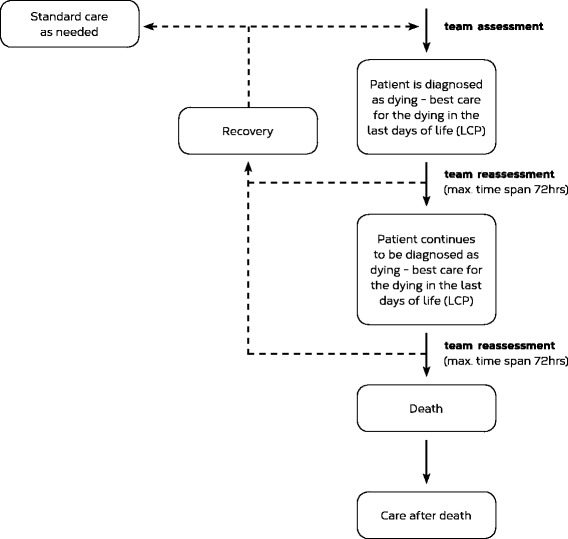
The trajectory of the Liverpool Care Pathway (LCP) [2, 44]. Routine interprofessional team (IPT) reassessment identifies patients who are no longer classified as dying

A strict requirement of the LCP is the regular reassessment of the diagnosis of the dying process [2]. Many palliative care professionals can describe anecdotal experiences, in which patients have stabilised during the dying phase and continued living for a period of time. Hence, despite being called “pathway”, the LCP is not designed as a one-way street with a linear trajectory, but provides decision points and loops (see Fig. 1).

How many patients recover while in the LCP? How often is the LCP discontinued in practice? Data answering these questions are lacking and have been considered a research priority [22, 32]. Consequently, we conducted an open cohort study to investigate the incidence, features and trajectory of patients who were discontinued from the LCP. The setting is a university hospital specialized palliative care unit (SPCU) in Germany. Our objective was to analyse the characteristics of LCP patients, including frequency for and reasons of LCP discontinuation, quality of life, and survival times of discontinued LCP patients. Our hypotheses were (1) it is common to discontinue patients from the LCP, (2) quality of life does not decrease for discontinued LCP patients, and (3) discontinued patients live longer (in comparison to the patients who stay on the LCP).

## Methods

We followed the STROBE guidelines for reporting observational cohort studies [33]. This study was approved by the ethics board of the Medical Faculty of Heinrich Heine University Düsseldorf (protocol number 5003, approved 02.03.2015). Given the retrospective and observational nature of our study, the ethics board waived the requirement for informed consent.

### Study design

**Fig. 2:**
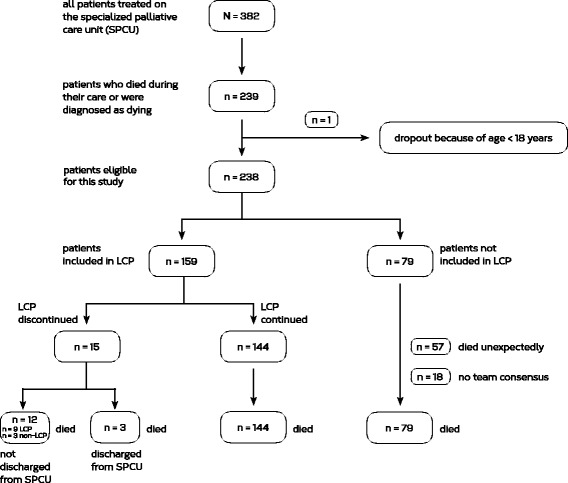
Flow chart of the cohort study design

We used an open cohort study design that is appropriate to follow a group of patients with different outcomes (death in LCP vs. LCP discontinued) over time. We assessed the survival time as primary outcome along with prognostication and a number of quality of life indicators as secondary outcomes. The cohort included all palliative care patients (age > 18) who were admitted to the 8-beds specialized palliative care unit (SPCU) at the University Hospital Düsseldorf, Germany, and who were diagnosed as dying between January 2013 and December 2014 (Fig. 2). Standard care involved careful assessment of symptom burden, development of an interprofessional treatment plan, treatment of symptoms on the physical, psychological, social and spiritual level, as well as daily team meetings for re-evaluation, and 24-hour specialist palliative care backup.

All patients were followed-up until their death, including those for whom the LCP was discontinued and who were discharged from the SPCU. Data were collected at SPCU admission (baseline, t1), at the time of entry into the LCP (t2) and at the time of LCP discontinuation (t3). At baseline, three actuarial estimation of survival prognostication tools were used to estimate the risk of death and for calculating potential confounding or moderation: Palliative Prognostic Index (PPI) [34], Palliative Prognostic Score (PaP-S) [35] and Palliative Performance Scale (PPS) [36]. Clinician’s prediction of survival was used to group patients along the care trajectory using four distinct expectation categories of palliative stages: rehabilitation phase, early end of life phase, late end of life phase and terminal phase [34, 37, 38]. These categories and the prognostication tools served to characterize the cohort and for statistical analysis. In the context of this study, these instruments were of negligible impact on the care the patients received. Additionally, quality of life was measured using validated instruments for daily living abilities: Karnofsky index [39], Eastern Cooperative Oncology Group (ECOG) scale [40], Barthel index [41] and activities and existential experiences of life scale (AEDL) [42].

Symptom burden was assessed by a palliative care nurse using single items with 5-point-Likert scales (0–4) for ten major symptoms in palliative care (*vigilance, delirium, restlessness, sweating, fatigue, nausea, vomiting, dyspnoea, coughing* and *itching*). *Constipation* was recorded as a dichotomous variable (0–1). *Pain* was measured on a numeric rating scale from 0 to 10 and assessed by self-rating if possible, or by a palliative care nurse. The sum of these scores provided the overall symptom score (range 0–51). Furthermore, selected items were grouped to four core domains of symptoms to generate sub-scores: psychological burden (*vigilance, delirium* and *restlessness*; 0–12); *nausea/vomiting* (0–8); *dyspnoea* (0–4) and *pain* (0–10) for longitudinal assessment of symptom burden over time (t1, t2, t3).

Patients who were diagnosed as dying according to all four criteria by Ellershaw and Ward [2] (patient becomes bedbound, semicomatose, able to take only sips of fluid and no longer able to take oral drugs) were included into the LCP (version 12 in German) [43, 44]. Decreased functional status over time and not having an acute reversible reason for their decline were two additional aspects considered by the interprofessional team (IPT). The patient assessment was documented and mutually agreed by a minimum of one physician and one palliative care nurse. We used an in-house developed digital version of the LCP, which was integrated into the digital hospital patient management system (Medico, Cerner, North Kansas City, MO, USA). Digital patient chart information and written team discussion notes were used to evaluate the reasons in which the LCP was discontinued. Qualitative thematic analysis was used to group qualitative findings.

### Statistical analysis

All statistical tests were performed using SPSS version 22.0 for Windows (IBM, Armonk, NY, USA). Demographic variables and psychometric scales are presented as median and range. Prior to further analysis, sample data was tested for homogeneity of variance using Levene’s test.

Possible confounding variables concerning the exclusion from the LCP were identified by analysing descriptive data. Differences in the subgroup medians (died in LCP vs. discontinued) greater than 15 % were considered as possible confounders. The respective measurements, i. e., PPI score, Karnofsky index, LCP duration, disease category (cancer/non-cancer), were controlled for their predictive value concerning the exclusion from the LCP by means of binary logistic regression analyses.

For comparison of time of measurement for the symptom burden sub scores (*psychological symptoms, nausea/vomiting, pain* and *dyspnoea*) a multivariate analysis of variance with repeated measurements using Pillai’s trace with the symptom burden subscores as the dependent variables and time of measurement as within-subjects-factor was conducted. Survival curves were obtained using the Kaplan-Meier method and comparisons between subgroups were calculated by Breslow test (generalised Wilcoxon test). For all tests *p* < 0.05 was considered statistically significant.

## Results

We observed SPCU patients within the LCP between January 2013 and December 2014. From a total of 382 patients who were treated at the SPCU, 239 patients died during the study period (62.6 %). One patient was excluded because he was too young. 238 patients were eligible cohort members, of those, 159 (67 %) patients were included in the LCP (Figure 2). The LCP patients form our final sample. The characteristics are shown in Table 1. Of the remaining 79 non-included patients, the majority died without prior team expectation of imminent death (57 patients, 72 %) and in some cases no IPT consensus on the diagnosis of dying was reached during the primary LCP assessment (18 patients, 23 %). The median time span between SPCU admission and LCP inclusion was 99 hours (range 0–766 hours). The median LCP duration for included patients was 78.9 hours (range 0–695 hours).

**Table 1:** Description of the sample

Attribute	Value
Number (*n*)	159
Patient characteristics	
Age (years)^a^	71 (28–97)
SPCU stay (hours)^a^	146 (3–985)
Gender	
Female	85 (53.5 %)
Male	74 (46.5 %)
Religion	*n* = 159
Roman-catholic	70 (44 %)
Evangelical	43 (27 %)
Muslim	7 (4 %)
Other or unknown	5 (2 %)
None	34 (21 %)
Advanced directives	*n* = 159
Patient will	69 (43 %)
Health care proxy	66 (42 %)
Disease	*n = 159*
Cancer	135 (85 %)
Gastrointestinal	29 (18 %)
Haematological	22 (14 %)
Lung	18 (11 %)
Urogenital	16 (10 %)
Gynaecological	14 (9 %)
Ear-nose-throat	6 (4 %)
Unknown primary	17 (11 %)
Other	13 (8 %)
Non-cancer	24 (15 %)
Multi-organ failure	7 (4 %)
Central nervous system	7 (4 %)
Cardiovascular	6 (4 %)
Other	3 (2 %)
Prognostic scores (on admission)	
PPI (0–15)	*n* = 78
1–5 (>3 weeks)	18 (23 %)
6–15 (<3 weeks)	60 (77 %)
PaP-S 30 days survival (0–17.5)	*n* = 79
>70 % (0–5.5)	13 (17 %)
30–70 % (6–11)	30 (38 %)
<30 % (11.5–17.5)	36 (46 %)
PPS (0–100 %)	*n* = 83
>50 %	4 (5 %)
30–50 %	37 (38 %)
10–20 %	42 (51 %)
Palliative stage	*n* = 132
Rehabilitation phase	3 (2.3 %)
Early end of life phase	34 (25.8 %)
Late end of life phase	81 (61.4 %)
Terminal phase	14 (10.6 %)
*Quality of life (on admission)*	
Karnofsky (0–100 %)	*n* = 76
<30 %	55 (72.4 %)
>30 %	21 (27.6 %)
Data not available	83 (52 %)
ECOG^a^ [0–5]	4 (1–4), *n* = 140
Barthel^a^ [0–100]	20 (0–95), *n* = 150
AEDL^a^ [0–36]	18 (3–36), *n* = 159
Symptom burden^a^	*n* = 159
Total score (0–51)	15 (5–30)
Psychological burden (0–12)	4 (0–12)
Dyspnoea (0–4)	2 (0–4)
Nausea/vomiting (0–8)	0 (0–7)
Pain (0–10)	3 (1–9)

^a^Median, range

### LCP discontinuation: rate and reasons

A considerable number of LCP patients (15 out of 159; 9.4 %) were discontinued from the LCP after routine reassessment. Table 2 compares the characteristics of the 144 patients who died on the LCP with the subgroup of 15 discontinued LCP patients. The IPT documented the reasons for LCP discontinuation as: *improved vigilance* (*n* = 9), *eating and drinking again* (*n* = 8), *general functional improvement* (*n* = 6), *patient communicates again* (*n* = 6), *regained ability to swallow* (*n* = 4), *patient interacts with family* (*n* = 2), *team opinion equivocal* (*n* = 2) and *can leave the bed again* (*n* = 1).

**Table 2:** Comparison of patients who died during LCP care vs LCP-discontinued patients

	Died	Discontinued
Number (*n*)	144	15
No IPT consensus on LCP	-	2 (13 %)
*Patient characteristics*		
Age (years)^a^	69.5 (28–97)	74 (51–87)
SPCU^b^ stay (hours)^a^	130 (3–941)	355 (52–985)
Time on LCP (hours)^a,c^	22.5 (0–240)	46 (12–143)
Gender		
Female	78 (54 %)	7 (47 %)
Male	66 (46 %)	8 (53 %)
*Disease*		
Cancer	124 (86 %)	11 (73 %)
Non-cancer	20 (14 %)	4 (27 %)
*Prognostic scores* ^*d*^		
PPI	*n* = 68	*n* = 10
1–5 (>3 weeks)	14 (20.6 %)	4 (26.7 %)
6–15 (<3 weeks)	54 (79.4 %)	6 (60 %)
PaP-S	*n* = 72	*n* = 7
>70 % (0.5–5.0)	12 (16.7 %)	1 (14.3 %)
30–70 % (6.0–11.0)	27 (37.5 %)	3 (42.9 %)
<30 % (11.5–17.5)	33 (45.8 %)	3 (42.9 %)
PPS	*n* =73	*n* = 10
100–60 %	2 (2.7 %)	2 (20 %)
50–30 %	35 (47.9 %)	2 (20 %)
10–20 %	36 (49.3 %)	6 (60 %)
Palliative stage	*n* = 120	*n* = 12
Rehabilitation phase	2 (1.7 %)	1 (8.3 %)
Early end of life phase	30 (25.0 %)	4 (33.3 %)
Late end of life phase	75 (62.5 %)	6 (50 %)
Terminal phase	13 (10.8 %)	1 (8.3 %)
*Quality of life* ^*d*^		
Karnofsky	*n* = 69	*n* = 7
>30 %	20 (29 %)	1 (14 %)
<30 %	49 (71 %)	6 (86 %)
ECOG (0–5)^a^	4 (1–4), *n* = 129	4 (3–4), *n* = 11
Barthel (0–100)^a^	15 (0–95), *n* = 138	20 (0–70), *n* = 12
AEDL (0–36)^a^	18 (3–36), *n* = 144	19 (8–35), *n* = 15
Total Symptom score (0–51)^a^	15 (5–30), *n* = 144	14 (8–24), *n* = 15

^a^Median (range)

^b^Specialized palliative care unit

^c^First period of LCP care (if discontinued and reincluded later)

^d^On admission

None of our ancillary analyses concerning confounding or moderation of LCP exclusion showed significant predictive values (all *p* values > 0.05). The test statistics and p-values of the binary logistic regression analyses are shown in Table 3.

**Table 3:** Test statistics of the binary logistic regression analyses^a^

	B (*SE*)	*R* ^2^	*χ* ^*2*^	df	*p*	exp(B)	CI (95 %) for exp(B)
Disease category	0.81 (0.63)	0.01	1.66	1	0.198	2.26	0.68–7.78
Karnofsky index	0.36 (0.45)	0.01	0.65	1	0.421	1.43	0.60–3.42
PPI score	−0.23 (0.12)	0.45	3.67	1	0.055	0.79	0.62–1.01
LCP duration	0.01 (0.01)	0.02	3.72	1	0.054	1.01	1.00–1.02

R^2^ (Cox & Snell), *χ* ^*2*^ Wald-Test

^a^Binary regression analysis was performed by adding all variables into the block simultaneously (method = Enter)

Nine of the 15 patients re-entered the LCP at a later point (median interval until reinclusion 188 hours; range 16–602 hours; second LCP duration: median 11 hours; range 6–195 hours). Three patients died without re-entering the LCP and three patients were discharged from the hospital.

### Survival time of discontinued patients

**Fig. 3:**
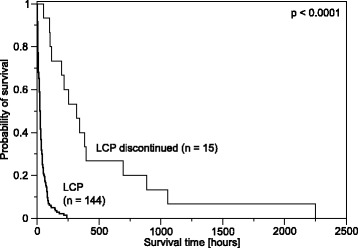
Kaplan-Meier estimates of survival for patients who were discontinued from the LCP compared with patients who were not discontinued from the LCP. Patients who recovered during the LCP period of care and were discontinued lived significantly longer than patients who did not recover and stayed on the LCP (*p* < 0.0001). Starting point for this analysis is the beginning of the LCP

Taking LCP initiation as the starting point, median survival time for the 15 discontinued patients was 318 hours (95 % confidence interval, CI, 158.94 to 477.06) and 22 hours for the 144 patients who stayed on the LCP (95 % CI 18.47 to 25.53). Figure 3 shows the Kaplan-Meier survival plot of the survival time for all LCP patients, comparing patients whose LCP care was discontinued to patients who stayed on the LCP. The overall survival time difference was significant in a Breslow test (*χ*^*2*^(1) = 26.85; *p* < 0.0001).

### Quality of life over time

**Fig. 4:**
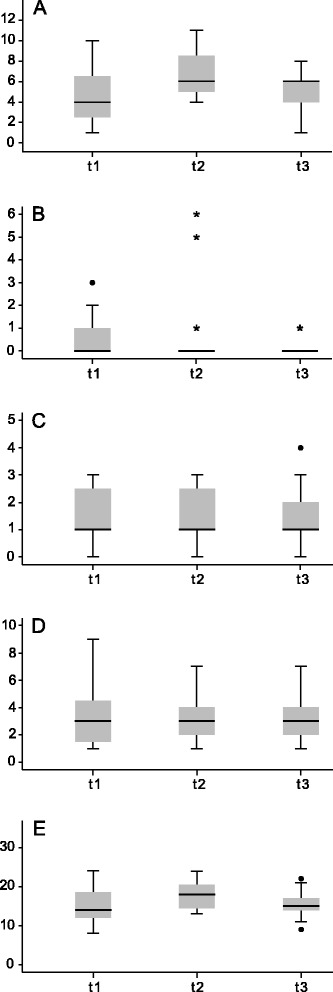
Symptom burden over time. The box plots show the distribution of symptom burden of patients who were first included and then discontinued from the LCP (n = 15). Patient were assessed at baseline (admission to the specialized palliative care unit) (t1), upon LCP inclusion (t2) and when discontinued from the LCP (t3). A, psychological burden (0–12); B, nausea and vomiting (0–8); C, dyspnoea (0–4); D, pain (0–10); E, total symptom burden (0–51). There was no significant change in the symptom burden subscores during LCP care (*V* = 0.56; *F* (8,7) = 1.11; *p* = 0.45). • = outliers; * = extreme values

In the dying phase, symptom burden is a good surrogate indicator for health-related quality of life [45–47]. We assessed the accumulated symptom burden for twelve different symptoms (vigilance, delirium, restlessness, sweating, fatigue, nausea, vomiting, dyspnoea, coughing, itching, constipation and pain) for three time points (t1, admission on ward; t2, LCP entry; t3, LCP discontinuation). To analyse the symptom burden in detail, we assessed four core domains of symptom burden (psychological distress, nausea/vomiting, dyspnoea and pain) over time for those 15 patients who were discontinued from the LCP (Fig. 4). A multivariate analysis of variance with repeated measurement using Pillai’s trace showed no significant change of the symptom burden during LCP care (*V* = 0.56; *F* (8,7) = 1.11; *p* = 0.45).

## Discussion

Diagnosing dying is a complex issue and sometimes a mystery [34, 48, 49]. We hypothesised that if applied adequately, it is a common finding to discontinue patients from an integrated care pathway for dying patients such as the LCP because they seem to be no longer diagnosed as dying. This hypothesis was confirmed. Given that discontinuation nearly exclusively equals evidence of performance improvement in those patients, we further hypothesised that discontinued patients live longer than those who stay on the LCP. This hypothesis was confirmed as well. We found no evidence for a decline in quality of life after discontinuation of the LCP.

The majority of discontinued patients (*n* = 9; 60 %) re-entered LCP care at a later point in time during the same admission period, while three patients died without prior re-diagnosing of dying by the IPT. In this second LCP care, patients died after a short period of time (median 11 hours). This finding demonstrates that even those, whose LPC care was discontinued, were indeed at a very late stage of their life. However and importantly, those three patients who were discharged after discontinuation (<2 % of total LCP sample) lived on for up to 13 weeks, which suggests that in a small proportion of patients diagnosing dying remains uncertain, even in an interprofessional, highly experienced team.

To our knowledge, this is the first study in the context of recent palliative care literature reporting on instances where “patients predicted as imminently dying have not died within that care episode” [32] and, therefore, is a direct response to the call for urgently needed research in the field of end-of-life care.

Anecdotal evidence has hinted towards a ten percent proportion of patients who “may appear briefly to be dying and then rally to have more time for a variety of reasons, most of which are social, emotional or spiritual.” [48]. We were able to confirm this number in a large cohort. The proportion reflects the inherent uncertainties in prognostication of death in which equivocal evaluation seems to be common even in highly experienced teams [34, 50].

We were able to include two thirds of our dying patients into the LCP. This is a higher rate than in a recent systematic review by Stocker and Close where an average uptake of the LCP of 47.4 % was reported [51]. The uptake ranges from 34–87 % [12]. Those patients who were not included in the LCP died a sudden death without prior signs or common phenomena of a terminal phase [52] as identified by the assessing palliative care team. A small subgroup of patients was not included into the LCP due to missing consensus between IPT members, demonstrating the relevance of active interprofessional team communication.

Our data show a benefit and necessity for routine IPT reassessment during care for the dying. This assessment is an important contributing factor in diagnosing non-dying patients in the LCP [53]. Interprofessional teamwork has received increasing attention within healthcare and care for the dying in particular [17, 53]. However, it takes time to control symptoms, ensure good communication and support families within a team approach [9]. It takes time to fully assess the patient system and to decide on a treatment plan [25]. Additionally, qualitative data suggests that integrated care plans can only be as effective as the degree of routine background accessibility of expert opinion for difficult cases [53]. Our study supports the notion that quality interprofessional communication improves care of the dying by stressing the association between rigorous and routine team discussion and the potential effect on diagnostic accuracy and survival time. Our data can serve as an indicator for the degree of uptake of integrated care plans in end-of-life care and reasons for non-inclusion or discontinuation. The question remains whether 10 % of misdiagnosing of dying is a high or low value in this context. Those numbers might serve as helpful in the development of quality criteria and for advanced care planning conversations in palliative care [54].

### Limitations

This study has several limitations. Non-controlled observational studies provide associations but no robust evidence of cause-effect relations. We found no statistical indication for confounding or moderation in our data. However, a trend was observed for the predictive value of the PPI score and the duration of LCP care justifying further analysis in future research.

Our study was set in a SPCU although the LCP was primarily designed for non-specialized settings [2]. The context of our study therefore needs to be adequately taken into consideration in future research studies. On the other hand, studying integrated care plans in a ‘gold standard’ setting can also provide valuable insights into the impact of integrated care plans on quality improvement, even in highly experienced palliative care teams [55].

## Conclusions

Diagnosing as dying and commencing an integrated end-of-life care plan (such as the LCP) does not result in a one-way route to death. Instead, if applied according to best practice, it is common to see patients stabilising or recovering during LCP care. We find that with 9.4 % of our LCP patients, the use of the integrated care plan was discontinued. Regular interprofessional assessment is important to identify those patients who stabilise during this period of care. Our findings provide no evidence for harm to patients cared along correctly applied LCP recommendations. The decision to discontinue the LCP upon re-assessment is typically correct and discontinued LCP patients seem to live longer as compared to patients who stay on the LCP. We conclude that informed and correct application of the LCP is a useful and safe means of good care for the dying.

## Abbreviations

CI: Confidence interval
IPT: Interprofessional team
LCP: Liverpool care pathway
SPCU: Specialized palliative care unit
